# Risk stratification for prediction of locoregional recurrence in patients with pathologic T1–2N0 breast cancer after mastectomy

**DOI:** 10.1186/s12885-020-07594-7

**Published:** 2020-11-23

**Authors:** Jianyang Wang, Yu Tang, Hao Jing, Guangyi Sun, Jing Jin, Yueping Liu, Yongwen Song, Weihu Wang, Hui Fang, Bo Chen, Shunan Qi, Hua Ren, Ning Li, Yuan Tang, Ningning Lu, Yong Yang, Zihao Yu, Shulian Wang, Yexiong Li

**Affiliations:** Department of Radiation Oncology, National Cancer Center/National Clinical Research Center for Cancer/Cancer Hospital, Chinese Academy of Medical Sciences & Peking Union Medical College, Beijing, 100021 China

**Keywords:** Breast cancer, Mastectomy: Locoregional recurrence, Radiotherapy, Risk stratification

## Abstract

**Background:**

Previous studies have revealed that nearly 15–20% of selected high-risk T1–2N0 breast cancers developed LRR after mastectomy. This study is aim to indentify the risk factors of locoregional recurrence (LRR) in patients with pathologic T1–2N0 breast cancer after mastectomy in a real-world and distinguish individuals who warrant postmastectomy radiotherapy (PMRT).

**Methods:**

Female patients treated from 1999 to 2014 in National Cancer Center of China were retrospectively reviewed. A competing risk model was developed to estimate the cumulative incidence of LRR with death treated as a competing event.

**Results:**

A total of 4841 patients were eligible. All underwent mastectomy plus axillary nodes dissection or sentinel node biopsy without PMRT. With a median follow-up of 56.4 months (range, 1–222 months), the 5-year LRR rate was 3.9%.Besides treatment era, age ≤ 40 years old (*p* < 0.001, hazard ratio [HR] = 2.262), tumor located in inner quadrant (*p* < 0.001, HR = 2.236), T2 stage (*p* = 0.020, HR = 1.419), and negative expressions of estrogen receptor (ER) and progesterone receptor (PR) (*p* = 0.032, HR = 1.485), were patients-related independent risk factors for LRR. The 5-year LRR rates were 1.7, 3.5, and 15.0% for patients with zero, 1–2, and 3–4 risk factors (*p* < 0.001).

**Conclusions:**

Risk Stratification based on age, T stage, ER/PR status and tumor location can stratify patients with pT1–2 N0 breast cancer into subgroups with different risk of LRR. PMRT might be suggested for patients with 3–4 risk factors.

## Background

Breast-conserving surgery plus radiotherapy and modified radical mastectomy are two of the standard surgical options for primary treatment of early-stage invasive breast cancers with primary tumor ≤5 cm and node-negative (ie, T1–2, N0 classification) [1, 2], achieving > 90% of 10-year overall survival (OS) rates and < 10% of 10-year locoregional recurrence (LRR) rates [3–5]. However,T1–2N0 breast cancer is a heterogeneous disease with different subgroups that demonstrate significant variation in risk for recurrence and survival [6]. Several studies have revealed that nearly 15–20% of selected high-risk T1–2N0 breast cancers developed LRR after mastectomy, which was comparable to or even higher than that with 1 to 3 positive nodes [7–12].

For the entire population of patients with T1–2N0 disease, previous studies showed 5-year LRR risk were only 3–6% in patients undergoing mastectomy and axillary clearance [9–11, 13]. Systemic review showed that postmastectomy radiotherapy (PMRT) reduced LRR risk from 6 to 2% at 5 years, and the absolute gain of 4% didn’t translate into 15-year survival benefit [13]. Thus, the indication of PMRT is debated in T1–2N0 breast cancer unless surgical margins are positive. However, it was stated that for every four local recurrences avoided in the first 5 years, one breast cancer death could be prevented [13]. Predicting the chance of local recurrence will allow selective use of PMRT in individual patients.

This study is to establish a local recurrence risk stratification model for T1–2N0 breast cancer after mastectomy and identify the subgroup where PMRT is indicated.

## Methods

### Patients

**Fig. 1:**
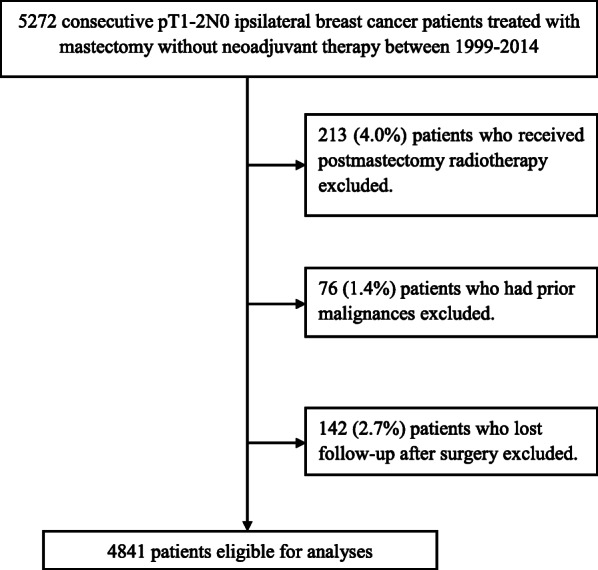
Flow diagram of included patients

We retrospectively reviewed the patients with invasive breast cancer who underwent mastectomy and axillary dissection or sentinel node biopsy without radiotherapy between January 1999 and April 2014 in National Cancer Center/Cancer Hospital of the People’s Republic of China. The hospital provides medical service to patients mainly from Northern and Northeast China, where lived 23.1% of population of China mainland. Eligibility criteria were as follows: female, age ≥ 18 years, no supraclavicular or internal mammary node metastasis, no distant metastasis, no neoadjuvant systemic therapy, complete resection (margins ≥1 mm), histologically confirmed primary tumor ≤5 cm with negative axillary nodes. The exclusion criteria were as follows: bilateral invasive breast cancer, previous history of malignancy except for nonmelanoma skin cancer or cervical carcinoma in situ, undergone postoperative radiotherapy and follow-up less than 1 month after surgery. A total of 4841 (97.2%) patients were eligible for analyses (Fig. 1).

The complete medical records of eligible patients were reviewed, and follow-up data were obtained from hospital records or from correspondence directly with the patient or their family. The following data were collected, including treatment era,age, gender, body mass index (BMI), tumor characteristics (diagnosis date; tumor size, histology, and grade;lymphovascular invasion (LVI) and nodal status; expressions of estrogen receptor [ER] and progesterone receptor [PR]; and human epidermal growth factor receptor 2 [Her2], and treatment specifics (type of definitive surgery, chemotherapy, targeted therapy, and endocrine therapy). In this study, we defined the status of ER/PR based on the pathologic report: ER/PR positive as + ~ +++ before 2003, or ≥ 1% expression of ER/PR after 2003 [14]. Tumors were considered Her-2-positive only if they had a +++ or ++ IHC score and positive Her-2 gene amplification based on fluorescence in situ hybridization (FISH). As data of Ki-67 was only available in 52.5% of patients, we defined the molecular subtype as: (1) Luminal (ER/PR+), (2) Her2 overexpression (Her2+, ER/PR-), (3) triple negative (ER/PR-, Her2-) groups. All patients were offered guideline-based surgery and adjuvant chemotherapy during the study period as per published recommendations. Trastuzumab was approved by the China Food and Drug Administration in September 2007, and it was subsequently administered to 233 (4.8%) patients out of entire study cohort according to new guideline after 2007 in China. Thus we divided the cohort by year of 2017 to explore the influence of treatment era.

### End points

After completion of adjuvant treatments, regular follow-up was conducted and continued until death or loss to follow-up. LRR was defined as reappearance of cancer in the ipsilateral breast or chest wall, axillary, supraclavicular fossa, infraclavicular fossa, or internal mammary nodes (IMN), irrespective of distant metastasis. Distant failure was defined as any evidence of metastatic disease beyond the locoregional regions mentioned above. LRR, distant metastasis-free survival (DMFS), disease-free survival (DFS), and overall survival (OS) rates were calculated from the date of the definitive surgery using the Kaplan–Meier method. Differences in survival were tested by log-rank test. The cumulative incidences of LRR were investigated via competing risk analysis methods of Fine and Gray. The competing event for the cumulative incidence was death without the event of interest.

### Statistical analysis

Patients’ demographic and clinicopathological characteristics were summarized through descriptive analysis. Continuous variables were described as means (SD) and compared using the Student’s *t*-test. Qualitative variables were described as frequencies and percentages and compared using the Fisher exact or χ^2^ test. Independent prognostic factors were identified using Cox stepwise regression analysis for variables with a *p*-value < 0.1 in univariate analysis. For missing information in some variables, e.g. tumor location and grade, those cases are excluded in univariate analysis. Patients were categoried based on the magnitude and differences of LRR between the subgroups with different number of independent risk factors for LRR. All *p-*values are two-tailed, and confidence interval [CI] were calculated at the 95% level. A *p-*value < 0.05 was considered statistically significant.

## Results

### Clinical characteristics

Table 1 summarizes the demographic, tumor, and treatment characteristics. The median age was 51 years (range, 19–89). Among the 3235 patients who received adjuvant chemotherapy, anthracycline- and / or paclitaxel-based regimens were used in 2627 (81.2%). A total of 3113 (98.8%) out of 3150 patients with positive ER/PR received endocrine therapy. The median duration of endocrine therapy was 46 months (1–160 months). A total of 233 (23.8%) out of 977 patients with HER-2-positive disease received trastuzumab.

**Table 1:** Baseline characteristics and 5-year LRR rates of 4841 pT1–2 N0 breast cancer patients after mastectomy

Variables	n (%)	5-year LRR (%)	p
Treatment era			
1999–2007	1915 (39.6)	5.5	< 0.001
2008–2014	2926 (60.4)	2.8	
Age (years)			
Median	51.1 ± 10.5		
≤ 40	723 (14.9)	7.5	< 0.001
> 40	4118 (85.1)	3.3	
Tumor quadrant			
Inner	1255 (25.9)	5.3	< 0.001
Non-inner	3486 (72.0)	3.0	
Unknown	100 (2.1)	18.9	
pT stage			
T2	1954 (40.4)	5.3	0.003
T1	2887 (59.6)	3.0	
Molecular subtype			
Luminal	3150 (65.0)	2.8	< 0.001
Her2 overexpression	501 (10.3)	5.0	
Triple-negative	1076 (22.2)	6.5	
Unknown	114 (2.4)	5.3	
LVI			
Yes	184 (3.8)	8.1	0.011
No	4631 (95.7)	4.0	
Unknown	26 (0.5)	11.6	
Tumor grade			
I and II	2803 (57.9)	3.3	0.012
III	1137 (23.5)	5.1	
Unknown	901 (18.6)	4.6	
Histology			
IDC	4399 (90.9)	4.0	0.356
ILC	141 (2.9)	1.9	
IMPC	26 (0.5)	0.0	
MBC	4 (0.1)	0.0	
Medullary carcinoma	56 (1.2)	6.0	
Other	215 (4.4)	2.4	
Adjuvant chemotherapy			
Yes	3236 (66.8)	2.1	< 0.001
No	1605 (33.2)	4.8	
Endocrine therapy			
Yes	3113 (64.3)	2.8	< 0.001
No	1682 (34.7)	6.1	
Unknown	46 (1.0)	0.0	
Median time (months)	44.7 ± 21.6		
Anti-Her2 target therapy			
Yes	233 (4.8)	3.4	0.372
No	4580 (94.6)	4.0	
Unknown	28 (0.6)	0.0	

*ER* Estrogen receptor, *Her2* Human epidermal growth factor receptor 2, *IDC* Invasive ductal carcinoma, *ILC* Invasive lobular carcinoma, *IMPC* Invasive micropapillary carcinoma, *LRR* Locoregional recurrence, *LVI* Lymphovascular invasion, *MBC* Metaplastic breast carcinoma, *PR* Progesterone receptor

### Patterns of LRR and survivals

With a median follow-up period for survivors of 55.2 months (range, 1–222 months), 234 LRR developed in 186 patients (3.8%) (LRR group), including 82 (44.1%) patients with isolated chest wall relapses, 81 (43.5%) with isolated regional lymph nodes (LN) relapses, and 23 (12.4%) with both chest wall and regional LN relapses (Table 2). The 5-year actuarial LRR rates were 3.9% for the entire cohort.

**Table 2:** Prevalence and sites of 234 locoregional recurrence in 186 patients

Sites of recurrence	Number	%*
Isolated chest wall	82	35.0
Chest wall + Regional LN	23	9.8
Chest wall + axillary LN + SC LN	3	1.3
Chest wall + SC LN + IMN	5	2.1
Chest wall + axillary LN	1	0.4
Chest wall + SC LN	6	2.6
Chest wall + IMN	8	3.4
Regional LN	81	34.6
Axillary LN + SC LN + IMN	2	0.9
Axillary LN + SC LN	6	2.6
SC LN + IMN	7	3.0
Axillary LN	16	6.8
SC LN	42	17.9
Internal mammary nodes	8	3.4

*IMN* Internal mammary nodes, *LN* Lymph nodes, *SCA* Supraclavicular;

*, percentage of total 234 locoregional recurrence

A total of 347 (7.2%) patients developed distant metastases, 99 (53.2%) in the 186 patients with LRR (LRR group) and 248 (5.3%) in the 4655 patients without LRR (non-LRR group). Among the 99 patients with both DM and LRR, 62 (62.6%) patients had concomitant LRR and DM, defined as LRR and DM occurred within 1 month. Death occurred in 205 (4.2%) patients, 56 (30.1%) in LRR group and 149 (3.2%) in non-LRR group.

For the entire cohort, the 5-year DMFS, DFS, and OS rates were 92.9, 91.1, and 96.4%, respectively. Compared with non-LRR group, LRR group suffered significant lower 5-year DMFS (53.5% vs. 94.8%, *p* < 0.001) and OS (75.9% vs. 97.4%, *p* < 0.001) from the initial surgery.

### Risk stratification and comparison of prognosis

**Fig. 2:**
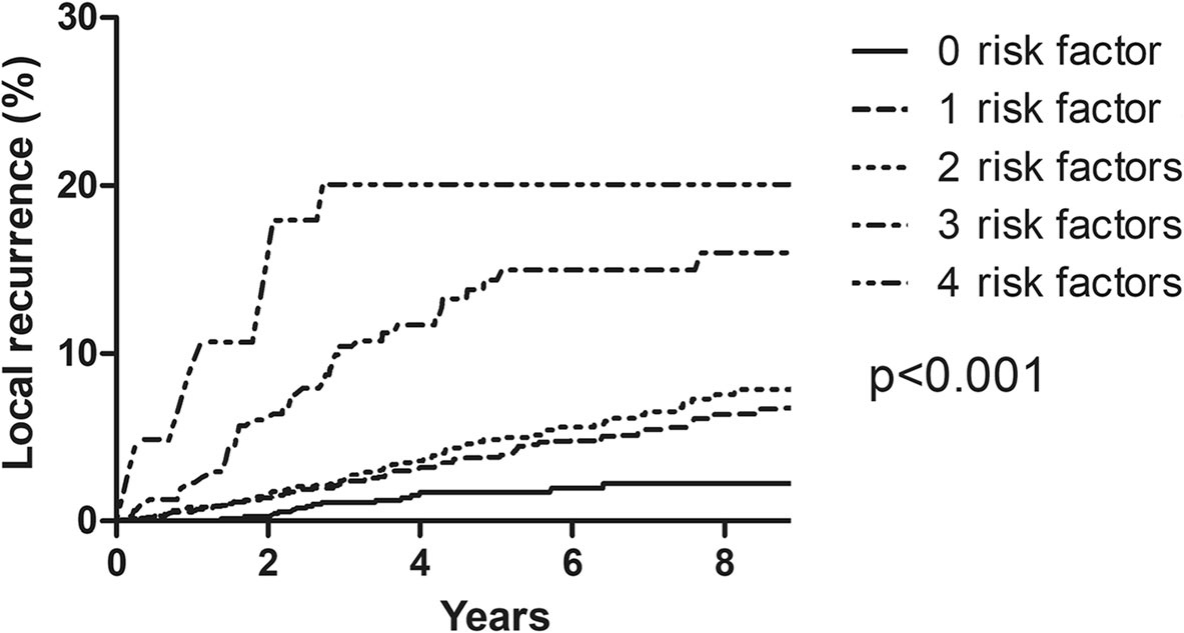
Competing-risks plots of the locoregional recurrence of all 4841 patients stratified by numbers of risk factors

**Fig. 3:**
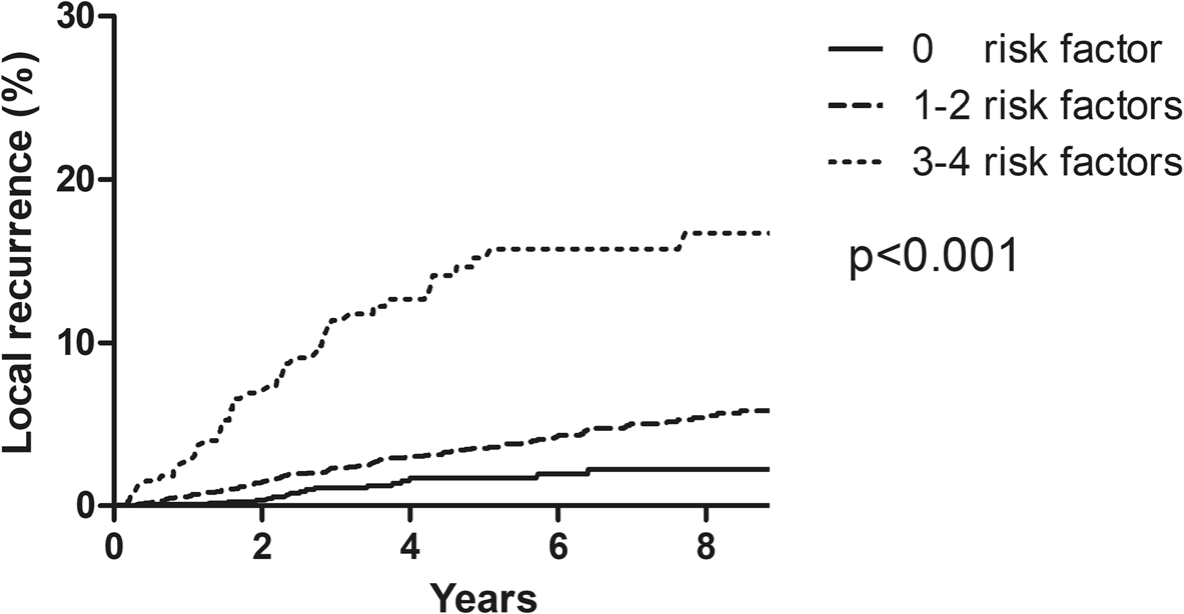
Competing-risks plots of the locoregional recurrence of all 4841 patients with risk stratification.

Univariate analysis of varialbles for LRR was summarized in Table 1. Table 3 showed the corresponding results of multivariate analysis. Because the difference in 5-year LRR between the Her2 overexpression and triple negative groups was not significant (5.0 vs. 6.5, *p* = 0.112), we combined these two groups into one group (ER/PR-) in multivariate analysis. Age ≤ 40 years old, ER/PR-, T2 stage, tumor located in inner quadrant and treatment era of 1999-2007 were independent risk factors for LRR. Patients were stratified into five subgroups according to age, ER/PR, T stage and tumor location, and 5-year LRR were 1.7, 3.4, 3.8, 14.4 and 20.0% for patients with 0, 1, 2, 3, and 4 risk factors, respectively (Fig. 2). Based on the magnitude and differences of LRR between the five subgroups, patients were further stratified into three groups: 1177 patients (23.8%) with zero risk factors, 3333 patients (67.5%) with 1–2 risk factors, and 331 patients (6.7%) with 3–4 risk factors. For patients with zero, 1–2, and 3–4 risk factors, the 5-year LRR rates were 1.7%, 3.5%, and 15.0%, respectively (*p* < 0.001) (Fig. 3).

**Table 3:** Significant prognostic factors for 5-year LRR by multivariate regression analyses

Variables	Multivariate analyses		
	LRR		
	HR	95%CI	*p*
1999–2007 vs. 2008–2014	1.923	1.380–2.688	< 0.001
Age ≤ 40 years old vs. > 40 years old	2.262	1.646–3.107	< 0.001
Inner location vs. Non-inner location	2.236	1.787–2.798	< 0.001
T2 stage vs. T1 stage	1.419	1.061–1.898	0.018
ER/PR (−) vs. ER and PR(+)	1.485	1.042–2.117	0.029
LVI vs. without LVI	1.053	0.879–1.262	0. 575
Grade III vs. Grade I and II	1.237	0.8861.726	0. 212
IDC vs. other pathology type	0.848	0.715–1.006	0.059
Adjuvant chemotherapy vs. non-adjuvant chemotherapy	1.429	0.951–2.147	0.086
Endocrine therapy vs. non- Endocrine therapy	0.728	0.524–1.012	0.059
Anti-Her2 target therapy vs. non- Anti-Her2 target therapy	0.606	0.246–1.492	0.276

*ER* Estrogen receptor, *Her2* Human epidermal growth factor receptor 2, *HR* Hazard ratio, *IDC* Invasive ductal carcinoma, *LRR* Locoregional recurrence, *LVI* Lymphovascular invasion, *PR* Progesterone receptor

## Discussion

To the best of our knowledge, this study represents the largest series focusing on risk factors for LRR in pT1–2 N0 breast cancer. We have identified four independent prognostic factors which were associated with an increased risk of LRR after adjusting for treatment era, including age ≤ 40 years, tumors size over 2 cm, negative expression of ER/PR, and primary tumor located in inner quadrant. In contrast to the 5-year LRR rate of 3.9% described in the entire T1–2N0 population, 6.7% of entire group, who had 3 or more risk factors, suffered from an increased 5-year LRR rate of 15.0%, which surpasses the risk of recurrence documented for patients with one to three positive lymph nodes [10, 15, 16]. In addition, the LRR curve in the highest risk category plateaued after 5 years, which might attribute to large proportion of ER/PR negative tumors in this group. It has been shown that the risk of relapse for patients with triple-negative and HER2 positive tumors was largely confined to the first 5 years after diagnosis, whereas the risk for patients with ER/PR positive tumors continued for 20 years after 5 years [17].

Early breast cancer patients usually achieve a favorable prognosis. Yet, a small proportion of patients fail locoregionally in the modern treatment system. A few trials have demonstrated that subgroups of pT1-2N0 patients with multiple adverse risk factors were at higher risk of LRR [9, 11, 12, 18–21]. Although the risk factors which were associated with LRR have varied among different studies and time eras, the proportion of patients at high risk of LRR was usually around 5–15%. Abi-Raad et al. reported that for patients with three or more of the following risk factors: presence of LVI, close or positive margins, tumor size > 2 cm, age < 50 years and no systemic therapy, which accounted for 8.7% of the entire cohort, the 10-year LRR was 19.7% [9]. Jwa et al. showed patients with age < 50 years and no systemic therapy suffered a 10-year LRR of 13.5%, which accounted for 5.9% of the entire cohort [20]. Truong et al. showed women with pT1-2N0 breast cancer experienced a LRR risk of approximately 20% in the presence of Grade 3 disease with LVI or Grade 3 disease, T2 tumors, and no systemic therapy, which accounted for 12.6% of the entire cohort [11, 18]. Li et al. showed that the following three risk factors, including age ≤ 40 years, primary tumor size ≥4.5 cm and number of nodes resected ≤10, helped to identify 10.5% of patients among T1–2N0 breast cancer at high risk for LRR, and their 5-year LRR rate was 24.3% [18].

Interestingly, we found that medial tumor was independently associated with high risk of LRR. A few studies have consistently shown that medial breast cancers carry a worse prognosis than lateral breast cancers, even after adjusting for other known prognostic factors [22–26]. Since internal mammary nodal involvement occurred more frequently in patients with medially located tumors [27], coverage of internal mammary nodes in the radiation field might be considered.

The 5-year LRR rate for high risk group among pT1-2N0 breast cancer was over 14% [9, 11, 12, 18–21], which is even higher than those with 1 to 3 nodes metastasis [7–12]. Early Breast Cancer Trialists’ Collaborative Group reported that PMRT reduced LRR about two thirds, and for every four LRR avoided, one breast cancer death could be prevented. In node-negative breast cancer, PMRT resulted in a 4% absolute reduction on the 5-year LRR risk, but didn’t improve OS. The absolute LRR risk reduction was much smaller in node-negative compared with node-positive breast cancer (4% vs. 17.1%) [13]. This might explain why node-positive breast cancer, rather than node-negative, had OS benefit from PMRT. It seems that the higher the LRR risk, the more OS benefit can be achieved by PMRT, providing that the risk of distant metastasis be well controlled by modern systemic therapy. The St. Gallen recommendations indicate that patients with a 10-year LRR rate of 20% or more require PMRT [28]. Growing evidences together with the findings from the present study showed pT1–2 N0 breast cancer is a heterogeneous disease. A small proportion with 5-year LRR risk around 20% could be identified based on risk factors currently available on routine clinical practice [9–12, 21]. These subgroups might potentially benefit from PMRT.

Interpretation of the current study’s findings must be made cautiously in view of its limitations. First, the retrospective design has inherent biases in patient and treatment selection. However, observational studies allow examination of outcomes following non-selective patient care in the real world. Therefore, observational studies are relied on to provide additional information ranking the individual with different risk factors by their impact on LRR. Second, the median follow-up time of 55.2 months is relatively short and might impair the accuracy of survival and LRR estimation. Third, the LRR rate might be underestimated due to the retrospective design. Last, patients were diagnosed and treated over a long period,changes in the diagnosis and treatment of breast cancer might have affected patients’ prognoses. Therefore,we included treatment era as a confounder to adjust for the result of the analyses. This model is valid regardless of treatment era, because age, T stage, ER/PR status and tumor location were independent prognostic factors for LRR after adjusting for treatment era. However, patients treated after 2007 had significantly lower risk of LRR than before 2007, the proportion of high-risk subgroup will become smaller in current practice.

## Conclusion

Our study demonstrated risk model based on age, T stage, ER/PR status and tumor location can stratify patients with pT1–2 N0 breast cancer into subgroups with different risk of LRR. If validated, PMRT should be considered for high-risk subgroup.

## Data Availability

The data of this study is available only under the approval of Independent Ethics Committee of Cancer Hospital, Chinese Academy of Medical Sciences.

## Abbreviations

CI: Confidence interval
DFS: Disease-free survival
DMFS: Distant metastasis-free survival
ER: Estrogen receptor
Her2: Human epidermal growth factor receptor 2
IDC: Invasive ductal carcinoma
ILC: Invasive lobular carcinoma
IMN: Internal mammary nodes
IMPC: Invasive micropapillary carcinoma
LN: Lymph nodes
LRR: Locoregional recurrence
LVI: Lymphovascular invasion
MBC: Metaplastic breast carcinoma
OS: Overall survival
PMRT: Postmastectomy radiotherapy
PR: Progesterone receptor

## References

[1] Veronesi U, Cascinelli N, Mariani L, Greco M, Saccozzi R, Luini A, Aguilar M, Marubini E (2002). Twenty-year follow-up of a randomized study comparing breast-conserving surgery with radical mastectomy for early breast cancer. N Engl J Med.

[2] Litiere S, Werutsky G, Fentiman IS, Rutgers E, Christiaens MR, Van Limbergen E, Baaijens MH, Bogaerts J, Bartelink H (2012). Breast conserving therapy versus mastectomy for stage I-II breast cancer: 20 year follow-up of the EORTC 10801 phase 3 randomised trial. Lancet Oncol.

[3] Park EH, Min SY, Kim Z, Yoon CS, Jung KW, Nam SJ, Oh SJ, Lee S, Park BW, Lim W (2017). Basic facts of breast Cancer in Korea in 2014: the 10-year overall survival Progress. J Breast Cancer.

[4] Wang SL, Fang H, Song YW, Wang WH, Hu C, Liu YP, Jin J, Liu XF, Yu ZH, Ren H (2019). Hypofractionated versus conventional fractionated postmastectomy radiotherapy for patients with high-risk breast cancer: a randomised, non-inferiority, open-label, phase 3 trial. Lancet Oncol.

[5] Darby S, McGale P, Correa C, Taylor C, Arriagada R, Clarke M, Cutter D, Davies C, Ewertz M, Godwin J (2011). Effect of radiotherapy after breast-conserving surgery on 10-year recurrence and 15-year breast cancer death: meta-analysis of individual patient data for 10,801 women in 17 randomised trials. Lancet.

[6] Rowell NP (2009). Radiotherapy to the chest wall following mastectomy for node-negative breast cancer: a systematic review. Radiother Oncol.

[7] Khan AJ, Milgrom SA, Barnard N, Higgins SA, Moran M, Shahzad H, Kim S, Goyal S, Al-Faraj F, Kirstein L (2014). Basal subtype, as approximated by triple-negative phenotype, is associated with locoregional recurrence in a case-control study of women with 0-3 positive lymph nodes after mastectomy. Ann Surg Oncol.

[8] Mukesh MB, Duke S, Parashar D, Wishart G, Coles CE, Wilson C (2014). The Cambridge post-mastectomy radiotherapy (C-PMRT) index: a practical tool for patient selection. Radiother Oncol.

[9] Abi-Raad R, Boutrus R, Wang R, Niemierko A, Macdonald S, Smith B, Taghian AG (2011). Patterns and risk factors of locoregional recurrence in T1-T2 node negative breast cancer patients treated with mastectomy: implications for postmastectomy radiotherapy. Int J Radiat Oncol Biol Phys.

[10] Jagsi R, Raad RA, Goldberg S, Sullivan T, Michaelson J, Powell SN, Taghian AG (2005). Locoregional recurrence rates and prognostic factors for failure in node-negative patients treated with mastectomy: implications for postmastectomy radiation. Int J Radiat Oncol Biol Phys.

[11] Truong PT, Lesperance M, Culhaci A, Kader HA, Speers CH, Olivotto IA (2005). Patient subsets with T1-T2, node-negative breast cancer at high locoregional recurrence risk after mastectomy. Int J Radiat Oncol Biol Phys.

[12] Abdulkarim BS, Cuartero J, Hanson J, Deschenes J, Lesniak D, Sabri S (2011). Increased risk of locoregional recurrence for women with T1-2N0 triple-negative breast cancer treated with modified radical mastectomy without adjuvant radiation therapy compared with breast-conserving therapy. J Clin Oncol.

[13] Clarke M, Collins R, Darby S, Davies C, Elphinstone P, Evans V, Godwin J, Gray R, Hicks C, James S (2005). Effects of radiotherapy and of differences in the extent of surgery for early breast cancer on local recurrence and 15-year survival: an overview of the randomised trials. Lancet.

[14] Goldhirsch A, Wood WC, Gelber RD, Coates AS, Thurlimann B, Senn HJ (2003). Meeting highlights: updated international expert consensus on the primary therapy of early breast cancer. J Clin Oncol.

[15] Katz A, Strom EA, Buchholz TA, Thames HD, Smith CD, Jhingran A, Hortobagyi G, Buzdar AU, Theriault R, Singletary SE (2000). Locoregional recurrence patterns after mastectomy and doxorubicin-based chemotherapy: implications for postoperative irradiation. J Clin Oncol.

[16] Sharma R, Bedrosian I, Lucci A, Hwang RF, Rourke LL, Qiao W, Buchholz TA, Kronowitz SJ, Krishnamurthy S, Babiera GV (2010). Present-day locoregional control in patients with t1 or t2 breast cancer with 0 and 1 to 3 positive lymph nodes after mastectomy without radiotherapy. Ann Surg Oncol.

[17] Esserman LJ, Moore DH, Tsing PJ, Chu PW, Yau C, Ozanne E, Chung RE, Tandon VJ, Park JW, Baehner FL (2011). Biologic markers determine both the risk and the timing of recurrence in breast cancer. Breast Cancer Res Treat.

[18] Li JL, Lin XY, Zhuang LJ, He JY, Peng QQ, Dong YP, Wu JX (2017). Establishment of a risk scoring system for predicting locoregional recurrence in T1 to T2 node-negative breast cancer patients treated with mastectomy: implications for postoperative radiotherapy. Medicine (Baltimore).

[19] Hastings J, Iganej S, Huang C, Huang R, Slezak J (2014). Risk factors for locoregional recurrence after mastectomy in stage T1 N0 breast cancer. Am J Clin Oncol.

[20] Jwa E, Shin KH, Lim HW, Jung SY, Lee S, Kang HS, Lee E, Park YH (2015). Identification of Risk Factors for Locoregional Recurrence in Breast Cancer Patients with Nodal Stage N0 and N1: Who Could Benefit from Post-Mastectomy Radiotherapy?. PLoS One.

[21] Lin PH, Yeh MH, Liu LC, Chen CJ, Tsui YC, Su CH, Wang HC, Liang JA, Chang HW, Wu HS (2013). Clinical and pathologic risk factors of tumor recurrence in patients with node-negative early breast cancer after mastectomy. J Surg Oncol.

[22] Sarp S, Fioretta G, Verkooijen HM, Vlastos G, Rapiti E, Schubert H, Sappino AP, Bouchardy C (2007). Tumor location of the lower-inner quadrant is associated with an impaired survival for women with early-stage breast cancer. Ann Surg Oncol.

[23] Colleoni M, Zahrieh D, Gelber RD, Holmberg SB, Mattsson JE, Rudenstam CM, Lindtner J, Erzen D, Snyder R, Collins J (2005). Site of primary tumor has a prognostic role in operable breast cancer: the international breast cancer study group experience. J Clin Oncol.

[24] Yang J, Tang S, Zhou Y, Qiu J, Zhang J, Zhu S, Lv Q (2018). Prognostic implication of the primary tumor location in early-stage breast cancer: focus on lower inner zone. Breast Cancer.

[25] Wu S, Zhou J, Ren Y, Sun J, Li F, Lin Q, Lin H, He Z (2014). Tumor location is a prognostic factor for survival of Chinese women with T1-2N0M0 breast cancer. Int J Surg.

[26] Lohrisch C, Jackson J, Jones A, Mates D, Olivotto IA (2000). Relationship between tumor location and relapse in 6,781 women with early invasive breast cancer. J Clin Oncol.

[27] Chen RC, Lin NU, Golshan M, Harris JR, Bellon JR (2008). Internal mammary nodes in breast cancer: diagnosis and implications for patient management -- a systematic review. J Clin Oncol.

[28] Wolff AC, Hammond ME, Hicks DG, Dowsett M, McShane LM, Allison KH, Allred DC, Bartlett JM, Bilous M, Fitzgibbons P (2013). Recommendations for human epidermal growth factor receptor 2 testing in breast cancer: American Society of Clinical Oncology/College of American Pathologists clinical practice guideline update. J Clin Oncol.

